# Development of Predictive Nomograms for Clinical Use to Quantify the Risk of Amputation in Patients with Diabetic Foot Ulcer

**DOI:** 10.1155/2021/6621035

**Published:** 2021-01-14

**Authors:** Bocheng Peng, Rui Min, Yiqin Liao, Aixi Yu

**Affiliations:** ^1^Department of Orthopedic Trauma and Microsurgery, Zhongnan Hospital of Wuhan University, Wuhan 430071, China; ^2^Department of Endocrinology, Zhongnan Hospital of Wuhan University, Wuhan 430071, China; ^3^Department of Thyroid and Breast Surgery, Zhongnan Hospital of Wuhan University, 169 Donghu Road, Wuhan, Hubei, China

## Abstract

**Objective:**

To determine the novel proposed nomogram model accuracy in the prediction of the lower-extremity amputations (LEA) risk in diabetic foot ulcer (DFU).

**Methods and Materials:**

In this retrospective study, data of 125 patients with diabetic foot ulcer who met the research criteria in Zhongnan Hospital of Wuhan University from January 2015 to December 2019 were collected by filling in the clinical investigation case report form. Firstly, univariate analysis was used to find the primary predictive factors of amputation in patients with diabetic foot ulcer. Secondly, single factor and multiple factor logistic regression analysis were employed to screen the independent influencing factors of amputation introducing the primary predictive factors selected from the univariate analysis. Thirdly, the independent influencing factors were applied to build a prediction model of amputation risk in patients with diabetic foot ulcer by using R4.3; then, the nomogram was established according to the selected variables visually. Finally, the performance of the prediction model was evaluated and verified by receiver working characteristic (ROC) curve, corrected calibration curve, and clinical decision curve.

**Results:**

7 primary predictive factors were selected by univariate analysis from 21 variables, including the course of diabetes, peripheral angiopathy of diabetic (PAD), glycosylated hemoglobin A1c (HbA1c), white blood cells (WBC), albumin (ALB), blood uric acid (BUA), and fibrinogen (FIB); single factor logistic regression analysis showed that albumin was a protective factor for amputation in patients with diabetic foot ulcer, and the other six factors were risk factors. Multivariate logical regression analysis illustrated that only five factors (the course of diabetes, PAD, HbA1c, WBC, and FIB) were independent risk factors for amputation in patients with diabetic foot ulcer. According to the area under curve (AUC) of ROC was 0.876 and corrected calibration curve of the nomogram displayed good fitting ability, the model established by these 5 independent risk factors exhibited good ability to predict the risk of amputation. The decision analysis curve (DCA) indicated that the nomogram model was more practical and accurate when the risk threshold was between 6% and 91%.

**Conclusion:**

Our novel proposed nomogram showed that the course of diabetes, PAD, HbA1c, WBC, and FIB are the independent risk factors of amputation in patients with DFU. This prediction model was well developed and behaved a great accurate value for LEA so as to provide a useful tool for screening LEA risk and preventing DFU from developing into amputation.

## 1. Introduction

As a common epidemic in the 21st century, the prevalence of diabetes is exploding all over the world and becoming a major public health concern [1]. According to statistics, about 415 million people worldwide are known to have diabetes in 2015, and this number is still continuously growing, up to an estimated 642 million people by 2040 with a 55% increase in the next 20 years [2]. At the same time, as an inevitable result of the rapid increase in the number of people with diabetes, the incidence of diabetes complications has also presented a corresponding dramatic rise which put low-income and middle-income countries at the greatest risk of death [3, 4].

Based on the two main etiological factors of diabetic peripheral neuropathy and peripheral arterial disease (PAD), diabetic foot ulcer (DFU) is one of the most serious complications of diabetes, which makes a great contribution to most causes of nontraumatic lower-extremity amputations (LEA) and leads high mortality [5, 6]. It is reported that the long-term prognosis after LEA, which is closely related to DFU, is extremely poor, with a 3-year mortality rate ranging from 35% to 50% [7]. In the longer term, the overall 5-year mortality rate was even higher, ranging from 52% to 80% with major amputations and from 53% to 100% for those with any amputation [8].

DFU and their worst adverse consequences, especially amputation, have a catastrophic impact on the mental and physical health of patients, including prolonged hospitalization, heavy economic burden, difficult treatment, significantly impaired quality of life, and eventually lead to high mortality, making it urgent to propose an efficient strategy for prevention and treatment [9]. Efforts to prevent amputation therefore deserve more focuses, and it could be achieved by risk factor identification. Previous studies have shown that there are many significant risk factors for amputation in patients with diabetic foot, including long-term hyperglycemia, inflammatory markers, duration of diabetes, PAD, age, Wagner grade, and osteomyelitis [9]. Regrettably, there is no efficient predictive tool has been yet developed in this direction to estimate the risk of amputation in patient with DFU.

Considering these challenges, we tent to establish a predictive nomograms model to quantify the risk of amputation in patients with diabetic foot and to propose precautionary protocols. The nomogram can graphically represent the numerical relationship between specific disease and risk factors and intuitively predict the incidence of adverse events through a scoring system without any complicated calculation formula. From this point of view and based on previous study, we hope to provide a useful tool for clinicians to early identify and develop optimal treatment regimen for patients with diabetic foot to avoid unpleasant events such as amputation.

## 2. Methods and Materials

### 2.1. Study Design and Participants

In order to solve this clinical problem, we designed and implemented a retrospective study and 125 type 2 DM patients with diabetic foot ulcer who were hospitalized in Zhongnan Hospital of Wuhan University from January 2015 to December 2019 were included in this study at all. Among the 125 patients, 66 patients were from the Trauma and Microorthopaedics Center of Zhongnan Hospital of Wuhan University, and 59 patients were from the Diabetes Center of Endocrinology Department of Zhongnan Hospital of Wuhan University. The criteria for inclusion and exclusion were as follows: inclusion criteria: (1) all participating patients meet the type 2 DM diagnostic criteria issued by WHO (World Health Organization) in 1999 and the DFU diagnostic criteria issued by IDWGF in 2015, (2) the age of patients was over 18 years, and (3) all patients have informed consent to this study; exclusion criteria: (1) type 1 DM patients or secondary DM patients, (2) diabetic patients during pregnancy and lactation, (3) patients with other infections except DFU infection, (4) patients with malignant tumor, and (5) patients with severe lack of case data. This study has been approved by the Ethics Committee of Zhongnan Hospital of Wuhan University.

### 2.2. Data Collection

We designed the clinical investigation case report form (CRF) to collect the clinical data of the patients from the Hospital Information System (HIS) system of Zhongnan Hospital, including general demographic data such as sex, age, BMI, course of diabetes; history of diabetic complications, including diabetic retinopathy (DR), diabetic nephropathy (DN), and peripheral angiopathy of diabetic (PAD); and results of fasting venous blood biochemical examination for the first time after admission, including fasting blood glucose (FBG), glycosylated hemoglobin (HbA1c), white blood cells (WBC), red cell distribution width (RDW), total protein (TP), albumin (ALB), total bilirubin (TBIL), direct bilirubin (DBIL), total cholesterol (TC), triglyceride (TG), high density lipoprotein cholesterol (HDL-C), low density lipoprotein cholesterol (LDL-C), blood uric acid (BUA), and fibrinogen (FIB). According to the CRF, we design an epidata database to collect survey data, and in order to control the quality of data, all data were input and checked in parallel by two people.

### 2.3. Statistical Analysis

The data of continuous variables obeying normal distribution and nonnormal distribution are represented by “mean±standard deviation (*x*±*s*)” and “median (lower quartile, upper quartile) (M (P25p75)), respectively. Continuous variables that obey normal distribution are tested by *t*-test, and continuous variables that do not obey normal distribution are tested by nonparametric rank sum test (Wilcoxon). The categorical variables were expressed by percentage constituent ratio and chi-squared test used for comparison between groups. All the statistics were tested by two-side test, with *p* value less than 0.05 considered to be statistically significant. All confidence intervals (CI) are set to 95%. Primary predictive factors were submitted to single factor logistic regression analysis and multivariate logistic regression analysis to distinguish if they were influence factor or independent influence factors of amputation in patients with diabetic foot if their *p* values were less than 0.05 in the univariate analysis. Based on the independent influencing factors of amputation in patients with diabetic foot, we used the R4.2 software to establish a nomogram prediction model. Area under curve (AUC) of receiver operating characteristic curve was used to estimate the performance of the nomogram prediction model. The accuracy of the model was tested by the Hosmer-Lemeshow test, and a corrected calibration curve which includes 2000 bootstrap samples was used for the internal validation of the nomogram prediction model. The decision analysis curve (DCA) was employed to evaluate the clinical efficacy of the nomogram by analyzing the net benefit under different risk thresholds in patients with diabetic foot. All statistical analysis was carried out by using the SPSS26.0 and R4.2 software.

## 3. Results

### 3.1. Baseline Clinical Characteristics of Participants

Among the 125 DFU patients in the study, there were 22 patients with gangrene, 43 patients with severe infection, and 32 patients with severe PAD. Among these patients, there were 84 males and 41 females, of whom 58 (46.4%) underwent amputation (amputation group), with an average age of 63.86 ± 12.40 years old, and 67 (53.6%) without amputation (nonamputation group), with an average age of 62.76 ± 11.35 years. In the univariate analysis, the differences of the clinical data for 7 (course of DM, PAD, HbA1C, WBC, ALB, BUA, and FIB) of the 21 variables in the amputation group and nonamputation group were statistically significant (*p* < 0.05). There were no significant differences in age, sex, BMI, DR, DN, FBG, TP, RDW, TBIL, DBIL, TC, TG, HDL-C, and LDL-C between the two groups (*p* > 0.05),showed as Table 1.

### 3.2. Single Factor Logistic Regression Analysis

The variables (course of diabetes, PAD, WBC, HbA1C, BUA, ALB, and FIB) with *p* < 0.05 in univariate analysis were taken as the primary predictive factors. Taking amputation (assignment: without amputation = 0, amputation = 1) as dependent variable, the primary predictive factors (assignment: without PAD = 0, peripheral PAD = 1; other independent variables are linear introduction) as independent variables, single factor logistic regression analysis was carried out. The results showed that the course of diabetes, PAD, HbA1C, WBC, BUA, and FIB were the risk factors of amputation in patients with diabetic foot, while ALB was the protective factors of diabetic foot amputation, as shown in Table 2.

### 3.3. Multiple Factor Logistic Regression Analysis

**Figure 1:**
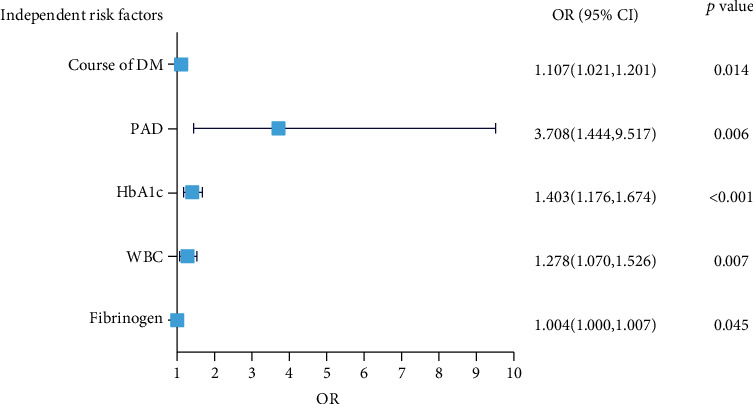
Plot of independent risk factors predicting the risk of amputation in patients with diabetic foot ulcer based on multivariate logistic regression analysis.

Taking the amputation of patients with diabetic foot as a dependent variable, then the seven independent variables (course of diabetes, PAD, HbA1C, WBC, ALB, BUA, and FIB) were putted into multiple factor logistic regression analysis. The inclusion and exclusion criteria of independent variables were 0.05. Forward stepwise regression was used to select the variables. The results showed that only five factors (the course of diabetes, PAD, HbA1C, WBC, and FIB) became independent risk factors of amputation in patients with diabetic foot (Figure 1).

### 3.4. Development of Nomogram Prediction Model

**Figure 2:**
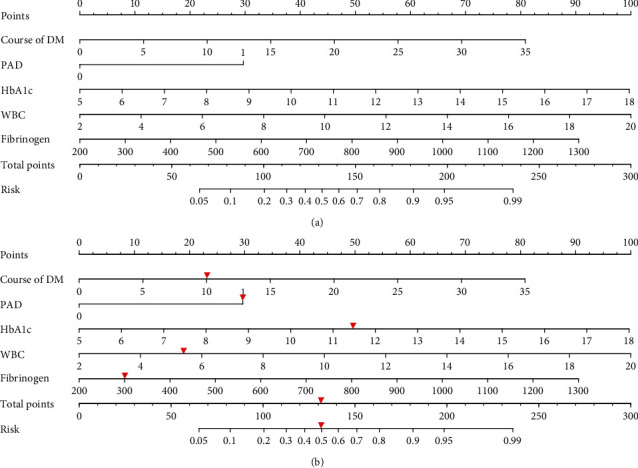
Development of the risk nomogram (a) and the dynamic nomogram for an example (b). The nomogram to predict the risk of amputation in patients with diabetic foot ulcer was developed with the predictors including course of diabetes, PAD, HbA1C, WBC, and FIB.

According to the results of multivariate logistic regression analysis, the five independent influence factors (course of diabetes, PAD, HbA1C, WBC, and FIB) of amputation in patients with diabetic foot ulcer were introduced to the R4.2 software; then, a nomogram model to predict the risk of amputation in patients with diabetic foot ulcer was established and is presented in Figure 2(a). The score corresponding to different values of each factor showed as Table 3, the total score of each factor is calculated, the total score ranges from 65 to 236, and the corresponding risk rate ranges from 0.05 to 0.99; the higher the total score, the greater the risk of amputation in patients with diabetic foot ulcer. Taking an example to better understand the nomogram model, if the patient with diabetic foot ulcer is complicated with PAD, diabetes course of 10 years, HbA1c of 11.5%, WBC of 5.67 ∗ 10^9^/L, and FIB of 301 mg/dL, the probability of amputation is estimated to be 49.4% (Figure 2(b)).

### 3.5. Validation of Nomogram Prediction Model

**Figure 3:**
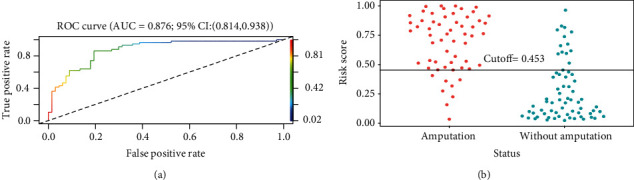
(a) The accuracy of the nomogram for predicting DFA using ROC curve. (b) The use of a cutoff value of 0.453 of the nomo-score performed well for the differential diagnosis of amputation from without amputation in the patients with diabetic foot ulcers.

**Figure 4:**
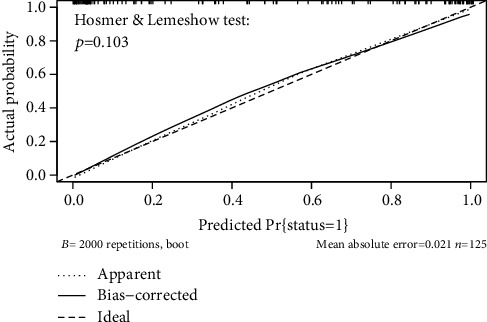
Calibration curves of the amputation risk in nomogram prediction. The thick dotted line represents an ideal prediction, and the thin dotted line represents the predictive ability of the nomogram. The closer the thick dotted line fit is to the thin dotted line, the better the predictive accuracy of the nomogram is.

**Figure 5:**
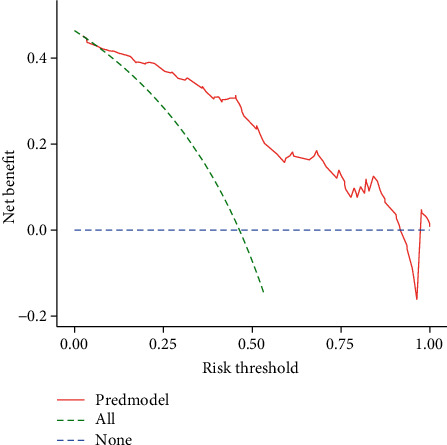
Decision curve analysis for the amputation risk nomogram. The *y*-axis measured the net benefit. The green dotted line represented the assumption that all patients had amputation. The blue dotted line represented the assumption that all patients had no amputation. The red solid line represented the risk nomogram.

The performance of the model in predicting amputation risk of patients with diabetic foot ulcer was assessed using receiver operating characteristic (ROC) curve (Figure 3(a)); the area under curve (AUC) of ROC was 0.876, which indicated the model had good performance. The optimum cutoff value for the risk of amputation in patients with diabetic foot ulcer was determined to be 0.453, and the true positive rate (TPR), true negative rate (TNR), and accuracy (ACC) value at optimum cutoff value were 0.86, 0.81, and 0.83, respectively. In Figure 3(b), the use of a cutoff value of 0.453 for risk of amputation in patients with diabetic foot ulcer made a fine play for the discrimination of amputation from without amputation in the participants. The Hosmer-Lemeshow test and corrected calibration curve which was done by producing 2000 bootstrap samples to displace the primary samples and repeating the whole modeling process were used to test the accuracy of the nomogram mode. The corrected calibration curve of the nomogram and the statistic data of Hosmer-Lemeshow test (*p* = 0.103) illustrated excellent degree of fit in Figure 4. In addition, in Figure 5, the DCA curve showed that the nomogram model was more practical and accurate when the risk threshold was between 6% and 91%. Based on the results of above validation, the nomogram of amputation prediction model of patients with diabetic foot ulcer revealed quite good predictive ability.

## 4. Discussion

As one of the common diabetes-related complications, diabetic foot ulcer (DFU) is affecting 10%-15% of people with diabetes and has a great influence on patients' quality of life due to its social and psychological consequences [3]. Because of DFU and secondary infection, most of our patients were living with nontraumatic lower-extremity amputations (LEA) and ultimately leads high mortality [10–12]. It is estimated that 17.4% to 27% of patients with PAD who have clinical manifestation progress to LEA within 5 to 18 years and the reported 5-year and 10-year survival rates after diabetic foot amputations were 40% and 24%, respectively, in a recent multinational cohort study [13–15]. Globally, the total healthcare expenditure related to DFU and LEA was very expensive, and this poses a significant strain on patients' families and national health care systems [16–18]. However, relevant tools that can effectively predict the risk of amputation in patient with DFU have rarely been studied. Therefore, it is meaningful to predict the risk of amputation considering the fatal adverse consequences of LEA in patients with DFU. Our purpose was to frame a risk score model that can be referred in clinical work to predict the risk of amputation in patients with DFU. To go further, our nomogram model can help clinicians identify high risk factors for amputation as early as possible so as to establish an appropriate treatment programs and take targeted measures to prevent morbidity and mortality.

In the previous literature, the amputation rates associated with diabetic foot disease range from 4.7 to 47.7% [19]. In the current study, we enrolled 125 type 2 DM with DFU patients, and LEA was present in 58 patients. To be more precisely, our findings also showed high rates of amputation; about 46.4% of the patients with DFU have been complicated with LEA, which is in accordance with other studies and commonly indicates a poor prognosis for patient with DFU. Through data review and analysis, we identified several independent risk factors that meaningfully associated with LEA, namely, as the course of diabetes, PAD, HbA1c, WBC, and fibrinogen, which were then collectively incorporated into the nomogram model to predict the risk of amputation and encouragingly founded it to be actively correlated with the LEA risk.

According to the statistical results in the previous research, the prevalence of PAD ranged from 8% to 56% [2, 20, 21]. In our study, the prevalence of PAD was 49.6% and roughly consistent with previous literature. Good blood circulation ensures the absorption of sufficient nutrients and the carrier of immune cells to play a role to prevent the infection of microorganisms. As a significant risk factor for the occurrence and adverse clinical outcomes of DFU, PAD produced harmful effects on wound healing to some extend and then leaded to amputation and eventually increased mortality rates if not timely treated [14]. Previous study has found that a 2.64 times increased risk of amputation in patients with PAD [20]. Many other similar studies have found that PAD is the potential cause of amputation [22–25]. This close connection between PAD and LEA enhances the necessity of vascular management and treatment for DFU, focused on appropriate strategies to promote wound healing and preserve limbs and life [26].

As we all know, HbA1c, as an indispensable gold standard for blood glucose control, can monitor and reflect the mean blood sugar over a period of 2-3 months. Long-term hyperglycemia exhibits an important role in poor wound healing and PAD, subsequently promotes susceptibility to infection, and glycemic control has been confirmed to reduce the rate of amputation combined with other prevention measures [27, 28]. A good glycemic control was found to be significant to prevent amputation in many studies. Studies have shown that intensive glycemic control could reduce 35% risk of amputations in patients with DFU [29]. According to a meta-analysis, every 1% increase in HbA1c was accompanied by a 26% increase in risk of LEA [30]. In this study, we demonstrated the association of higher HbA1c levels with worse incident LEA risk. All of the above results reinforce the importance of regular monitoring and treatment of hyperglycemia including lifestyle modification and compliance to medication to decrease the incidence of LEA [23, 31].

Infection was one of the most common causes of amputation, and inflammatory markers play a key role in the decision-making process of lower limb infection. Usually, clinicians use WBC count, ESR, and CRP to make a preliminary assessment of these patients or monitor the curative effect of medical or surgical treatment [32]. Ariz et al. observed that the count of WBC ≥ 15.0 × 10^9^/L was a predictor of amputation in patients with DFU, while ESR and CRP levels were only related factors. Infective marker in the form of total WBC count had been always deemed to be a predictor for LEA in DFU with almost one-third of our patients with leukocytosis upon presentation with progression to amputation [33]. Our present study further confirms that total WBC count was independent risk factors for LEA, and then, it is necessary to take measures to control inflammation in patients with DFU such as adequate debridement and effective anti-infective therapy.

In terms of the course of diabetes, Nazri Mohd Yusof et al. found the course of diabetes ≥ 10 years was positively correlated with the LEA risk and was momentous predictor of lower limb amputation in patients with DFU [34]. Previous studies by Lehto et al. and Zhang also confirmed this finding [35, 36]. Our study showed that the course of diabetes could be used to predict the risk of LEA; this was consistent with the above studies. In underdeveloped rural areas, patients who have diabetic foot complications were inclined to attend late to the hospitals due to inconvenient transportation. Delayed diagnosis and treatment of diabetic foot complications were closely related to increased amputation and mortality. Consequently, early diagnosis and prompt referral are of great significance for patients with DFU.

A convenient and efficacious marker of inflammation is fibrinogen, an acute phase protein, which was upregulated in patients with DFU and was associated with the severity of ulcers [37]. Study by Li et al. has shown that fibrinogen levels were found to be associated with acute-phase makers CRP levels, neutrophil, and WBC count, and fibrinogen values might be a valuable tool for assessing the disease severity and monitoring the disease progression in patients with DFU [38]. Apart from inflammatory reaction, fibrinogen may also produce an effect in endothelial injury, the formation of low permeability fibrin clot, thrombosis, abnormalities of blood flow, and platelet hyperactivity and accelerated the disease progression of PAD [39]. In our study, we demonstrated that fibrinogen was the independent risk factors of amputation in patients with DFU and suggested that it needed to be paid enough attention.

Our analysis demonstrated that the nomogram was well developed and showed a great accurate value for LEA prediction through discrimination capability evaluation. Consequently, the area under curve (AUC) of ROC was 0.876, which indicated the model had good performance. Then, the Hosmer-Lemeshow tested and corrected calibration curve which was done by producing 2000 bootstrap samples to displace the primary samples, and repeating the whole modeling process illustrated excellent degree of fit. In addition, the DCA curve showed that the nomogram model was more practical and accurate when the risk threshold was between 6% and 91%.

Above on those, our nomogram model exhibited a more accurate value for LEA prediction, and its construction greatly increased the clinical utility so as to dramatically improve the precision of the therapeutic options in clinical work.

To our best knowledge, this is the first study to determine risk factors of amputation for patients with diabetes foot using a novel proposed nomogram with excellent diagnostic accuracy, and the patient data for establishing the nomogram for predicting the risk of amputation for patients with DFU were from the Trauma and Microorthopaedics Center and Diabetes Center of Endocrinology Department, which means that the conclusions of our study are applicable not only to patients in orthopedic centers but also to patients with DFU in other internal medicine departments such as diabetes center. However, there are still several limitations with our current study. First, all patient data of this research came from one hospital; although the nomogram model achieved a preferable accuracy, it is still a large space for us to conduct a further prospective multihospital validation for confirming and improving the reliability of the nomogram to further increase the clinical utility. Second, this analysis is a retrospective design; not all clinicopathologic data is included in our study due to restricted data availability, and other potential risk factors need to be further included. Third, we only conducted the internal validation in this nomogram prediction model, and in the future, an external validation is also needed to improve the prediction value of the nomogram model.

In conclusion, after reviewing numerous studies and evaluating multiple variables, we demonstrated that the course of diabetes, PAD, glycosylated hemoglobin, WBC, and fibrinogen are the independent risk factors of amputation in patients with DFU. Based on these, we have established and validated a novel nomogram for the risk of LEA among patients with DFU. This model behaved a great accurate value and showed good discrimination for LEA prediction so as to help clinicians take targeted and active therapeutic regimen on medical interventions in time to prevent DFU from developing into amputation.

## Figures and Tables

**Table 1:** Differences in characteristics between the without amputation group and the amputation group.

Variables	Without amputation (*n* = 67)	Amputation (*n* = 58)	*T*/*Z* value	*p* value
Age (years)	62.91 ± 11.35	63.86 ± 12.40	-0.445	0.657
Gender (male/female)	46/21	38/20	0.139	0.709
BMI (kg/m^2^)	25.54 (23.36, 28.25)	25.40 (22.94, 29.15)	-2.500	0.803
Course of T2DM (years)	6.00 (4.00, 9.00)	10.00 (8.00, 15.25)	-4.680	<0.001
DR	19/48	25/33	2.963	0.085
DN	34/33	30/28	0.012	0.913
PAD	22/45	40/18	16.233	<0.001
FBG (mmol/L)	9.58 (6.54, 11.97)	9.60 (6.54, 13.00)	-0.451	0.652
WBC (10^9^/L)	7.02 (4.92, 8.19)	7.68 (6.11, 11.20)	-2.725	0.006
RDW (%)	13.40 (12.80, 14.30)	13.45 (12.78, 14.33)	-0.446	0.656
HbA1c (%)	8.77 ± 2.67	11.42 ± 2.45	1.288	<0.001
TP (mmol/L)	64.29 ± 6.91	62.60 ± 7.71	-1.550	0.121
ALB (g/L)	34.12 ± 6.15	30.37 ± 5.21	0.657	0.002
TBIL (*μ*mol/L)	9.00 (7.40, 12.00)	9.95 (7.65, 13.38)	-0.589	0.556
DBIL (*μ*mol/L)	2.00 (1.50, 2.70)	2.30 (1.20, 3.43)	-0.860	0.390
BUA (mmol/L)	313.20 (238.00, 376.00)	262.10 (208.35, 352.85)	-1.671	0.095
TC (mmol/L)	3.76 (3.09, 4.80)	3.36 (3.04, 4.22)	-1.767	0.077
TG (mmol/L)	1.01 (0.67, 1.59)	1.12 (0.84, 1.41)	-1.802	0.441
HDL-C (mmol/L)	1.02 ± 0.19	1.06 ± 0.29	-0.893	0.374
LDL-C (mmol/L)	3.19 ± 0.99	3.10 ± 2.27	0.279	0.632
FIB (mg/dL)	394.00 (330.00, 454.00)	480.50 (390.75, 578.00)	-3.767	<0.001

**Table 2:** Risk and protective factors of amputation in patients with diabetic foot ulcer.

Variables	*β*	SE	Wald	*p*	OR (95% CI)
Course of T2DM	0.105	0.033	10.441	0.01	1.111 (1.042, 1.184)
PAD	1.514	0.385	15.466	<0.001	4.545 (2.137, 9.667)
HbA1c	0.379	0.080	22.581	<0.001	1.460 (1.249, 1.707)
WBC	0.226	0.071	10.207	0.001	1.253 (1.091, 1.439)
ALB	-0.102	0.034	9.121	0.003	0.903 (0.846, 0.965)
FIB	0.005	0.002	9.821	0.002	1.005 (1.002, 1.008)

**Table 3:** Different index score, total score, and risk rate of nomogram.

Course of T2DM (years)	Point	PAD	Point	HbA1c	Point	WBC	Point	FIB	Point	Total points	Risk
0	0	Without PAD	0	5	0	2	0	200	0	65	0.05
5	12	PAD	30	6	8	4	11	300	8	82	0.10
10	23			7	15	6	22	400	16	100	0.20
15	35			8	23	8	33	500	25	113	0.30
20	46			9	31	10	44	600	33	123	0.40
25	58			10	38	12	56	700	41	132	0.50
30	69			11	46	14	67	800	49	141	0.60
35	81			12	54	16	78	900	58	151	0.70
				13	61	18	89	1000	66	163	0.80
				14	69	20	100	1100	74	182	0.90
				15	77			1200	82	198	0.95
				16	84			1300	91	236	0.99
				17	92						
				18	100						

## Data Availability

All the data are listed in the charts and attachments.

## References

[1] Schmidt A. M. (2018). Highlighting diabetes mellitus. *Arteriosclerosis, Thrombosis, and Vascular Biology*.

[2] Lavery L. A., Oz O. K., Bhavan K., Wukich D. K. (2019). Diabetic foot syndrome in the twenty-first century. *Clinics in Podiatric Medicine and Surgery*.

[3] Lin C. W., Armstrong D. G., Lin C. H. (2019). Nationwide trends in the epidemiology of diabetic foot complications and lower-extremity amputation over an 8-year period. *BMJ Open Diabetes Research & Care*.

[4] Font-Jiménez I., Llaurado-Serra M., Roig-Garcia M., De los Mozos-Perez B., Acebedo-Urdiales S. (2016). Retrospective study of the evolution of the incidence of non-traumatic lower-extremity amputations (2007–2013) and risk factors of reamputation. *Primary Care Diabetes*.

[5] Gherman D., Dumitrescu C. I., Ciocan A. N., Melincovici C. S. (2018). Histopathological changes in major amputations due to diabetic foot - a review. *Romanian Journal of Morphology and Embryology*.

[6] Hoffstad O., Mitra N., Walsh J., Margolis D. J. (2015). Diabetes, lower-extremity amputation, and death. *Diabetes Care*.

[7] Ugwu E., Adeleye O., Gezawa I., Okpe I., Enamino M., Ezeani I. (2019). Predictors of lower extremity amputation in patients with diabetic foot ulcer: findings from MEDFUN, a multi-center observational study. *Journal of Foot and Ankle Research*.

[8] Thorud J. C., Plemmons B., Buckley C. J., Shibuya N., Jupiter D. C. (2016). Mortality after nontraumatic major amputation among patients with diabetes and peripheral vascular disease: a systematic review. *The Journal of Foot and Ankle Surgery*.

[9] Peter-Riesch B. (2016). The diabetic foot: the never-ending challenge. *Endocrine Development*.

[10] Cramb S., Golledge J., Zhang Y., Lazzarini P. A. (2020). Trends in lower extremity amputation incidence in European Union 15+ countries 1990–2017. *European Journal of Vascular and Endovascular Surgery*.

[11] Mader J. K., Haas W., Aberer F. (2019). Patients with healed diabetic foot ulcer represent a cohort at highest risk for future fatal events. *Scientific Reports*.

[12] Jeyaraman K., Berhane T., Hamilton M., Chandra A. P., Falhammar H. (2019). Mortality in patients with diabetic foot ulcer: a retrospective study of 513 cases from a single centre in the Northern Territory of Australia. *BMC Endocrine Disorders*.

[13] Soo B. P., Rajbhandari S., Egun A., Ranasinghe U., Lahart I. M., Pappachan J. M. (2020). Survival at 10 years following lower extremity amputations in patients with diabetic foot disease. *Endocrine*.

[14] Wang N., Yang B. H., Wang G. (2018). A meta-analysis of the relationship between foot local characteristics and major lower extremity amputation in diabetic foot patients. *Journal of Cellular Biochemistry*.

[15] Jupiter D. C., Thorud J. C., Buckley C. J., Shibuya N. (2016). The impact of foot ulceration and amputation on mortality in diabetic patients. I: from ulceration to death, a systematic review. *International Wound Journal*.

[16] Carinci F., Uccioli L., Benedetti M. M., Klazinga N. S. (2020). An in-depth assessment of diabetes-related lower extremity amputation rates 2000–2013 delivered by twenty-one countries for the data collection 2015 of the Organization for Economic Cooperation and Development (OECD). *Acta Diabetologica*.

[17] Pedras S., Vilhena E., Carvalho R., Pereira M. G. (2020). Quality of life following a lower limb amputation in diabetic patients: a longitudinal and multicenter study. *Psychiatry*.

[18] Syed M. H., Salata K., Hussain M. A. (2020). The economic burden of inpatient diabetic foot ulcers in Toronto, Canada. *Vascular*.

[19] Weck M., Slesaczeck T., Paetzold H. (2013). Structured health care for subjects with diabetic foot ulcers results in a reduction of major amputation rates. *Cardiovascular diabetology*.

[20] Yazdanpanah L., Shahbazian H., Nazari I. (2018). Risk factors associated with diabetic foot ulcer-free survival in patients with diabetes. *Diabetes & Metabolic Syndrome: Clinical Research & Reviews*.

[21] Brownrigg J. R., Griffin M., Hughes C. O. (2014). Influence of foot ulceration on cause-specific mortality in patients with diabetes mellitus. *Journal of Vascular Surgery*.

[22] Sun J. H., Tsai J. S., Huang C. H. (2012). Risk factors for lower extremity amputation in diabetic foot disease categorized by Wagner classification. *Diabetes Research and Clinical Practice*.

[23] Aiello A., Anichini R., Brocco E. (2014). Treatment of peripheral arterial disease in diabetes: a consensus of the Italian Societies of Diabetes (SID, AMD), Radiology (SIRM) and Vascular Endovascular Surgery (SICVE). *Nutrition, Metabolism and Cardiovascular Diseases*.

[24] Gregg E. W., Li Y., Wang J. (2014). Changes in diabetes-related complications in the United States, 1990-2010. *The New England Journal of Medicine*.

[25] Gurney J. K., Stanley J., York S., Rosenbaum D., Sarfati D. (2018). Risk of lower limb amputation in a national prevalent cohort of patients with diabetes. *Diabetologia*.

[26] Kolossvary E., Farkas K., Colgan M. P. (2017). “No more amputations”: a complex scientific problem and a challenge for effective preventive strategy implementation on vascular field. *International angiology*.

[27] Hemmingsen B., Lund S. S., Gluud C., Vaag A., Almdal T. P., Wetterslev J. (2013). Targeting intensive glycaemic control versus targeting conventional glycaemic control for type 2 diabetes mellitus. *Cochrane Database of Systematic Reviews*.

[28] Fesseha B. K., Abularrage C. J., Hines K. F. (2018). Association of hemoglobin A1c and wound healing in diabetic foot ulcers. *Diabetes Care*.

[29] Hasan R., Firwana B., Elraiyah T. (2016). A systematic review and meta-analysis of glycemic control for the prevention of diabetic foot syndrome. *Journal of Vascular Surgery*.

[30] Adler A. I., Erqou S., Lima T. A., Robinson A. H. (2010). Association between glycated haemoglobin and the risk of lower extremity amputation in patients with diabetes mellitus-review and meta-analysis. *Diabetologia*.

[31] Farooque U., Lohano A. K., Rind S. H. (2020). Correlation of hemoglobin A1c with Wagner classification in patients with diabetic foot. *Cureus*.

[32] Reiner M. M., Khoury W. E., Canales M. B. (2017). Procalcitonin as a biomarker for predicting amputation level in lower extremity infections. *The Journal of Foot and Ankle Surgery*.

[33] Aziz Z., Lin W. K., Nather A., Huak C. Y. (2011). Predictive factors for lower extremity amputations in diabetic foot infections. *Diabetic Foot & Ankle*.

[34] Wukich D. K., Raspovic K. M., Suder N. C. (2017). Patients with diabetic foot disease fear major lower-extremity amputation more than death. *Foot & Ankle Specialist*.

[35] Lehto S., Rönnemaa T., Pyörälä K., Laakso M. (1996). Risk factors predicting lower extremity amputations in patients with NIDDM. *Diabetes Care*.

[36] Selby J. V., Zhang D. (1995). Risk factors for lower extremity amputation in persons with diabetes. *Diabetes Care*.

[37] Weigelt C., Rose B., Poschen U. (2009). Immune mediators in patients with acute diabetic foot syndrome. *Diabetes Care*.

[38] Li X. H., Guan L. Y., Lin H. Y. (2016). Fibrinogen: a marker in predicting diabetic foot ulcer severity. *Journal of Diabetes Research*.

[39] Farrell D. H. (2012). *γ*’ Fibrinogen as a novel marker of thrombotic disease. *Clinical Chemistry and Laboratory Medicine*.

